# Germline loss-of-function variant in the E3 ubiquitin ligase *TRAF2* in a young adult patient with medulloblastoma: a case report

**DOI:** 10.1186/s40478-024-01896-8

**Published:** 2024-12-20

**Authors:** Josh N. Vo, Andrea Franson, Sebastian M. Waszak, Yi-Mi Wu, Nicole Becker, Arul M. Chinnaiyan, Dan R. Robinson

**Affiliations:** 1 Michigan Center for Translational Pathology, University of Michigan, Ann Arbor, MI USA; 2 Department of Pathology, University of Michigan, Ann Arbor, MI USA; 3 Department of Pediatrics, University of Michigan, Ann Arbor, MI USA; 4 School of Life Sciences, École Polytechnique Fédérale de Lausanne, Lausanne, Switzerland; 5 Department of Neurology, University of California, San Francisco, San Francisco, CA USA

**Keywords:** Medulloblastoma, AYA, *TRAF2*, Genetic tumor syndromes

## Abstract

We identified a rare heterozygous germline loss-of-function variant in the tumor necrosis factor receptor-associated factor 2 (*TRAF2*) in a young adult patient diagnosed with medulloblastoma. This variant is located within the TRAF-C domain of the E3 ubiquitin ligase protein and is predicted to diminish the binding affinity of TRAF2 to upstream receptors and associated adaptor proteins. Integrative genomics revealed a biallelic loss of *TRAF2* via partial copy-neutral loss-of-heterozygosity of 9q in the medulloblastoma genome. We further performed comparative analysis with an in-house cohort of 20 medulloblastomas sequenced using the same platform, revealing an atypical molecular profile of the *TRAF2*-associated medulloblastoma. Our research adds to the expanding catalog of genetic tumor syndromes that increase the susceptibility of carriers to MB.

## Introduction

Medulloblastoma (MB) is an embryonal neoplasm of the cerebellum that primarily affects children, adolescents, and young adults, with an incidence rate of 5 cases per 1 million individuals [1]. Although the overall survival rate in many patients with localized disease and/or favorable molecular subgroups is high, the quality of life for patients with MB, in general, remains suboptimal [1, 2]. The long-term toxicity burden from current therapies underlines the urgent need for a deeper understanding of the biology of this malignancy to enable the development of more effective, less toxic therapies. Recent advancements in DNA methylation-based tumor profiling have unveiled the remarkable molecular heterogeneity of MB, identifying four distinct molecular subgroups: WNT (MB_WNT_), Sonic Hedgehog (MB_SHH_), Group 3, and Group 4 [1, 3]. These subgroups are characterized by unique genomic alterations and clinicopathological features, underscoring the importance of subgroup-specific therapeutic approaches [1, 3]. Elucidating the intricate molecular landscapes of each MB subgroup is imperative to identify novel therapeutic targets and optimize risk-stratified therapeutic strategies.

While MB is extensively documented within the pediatric population, there remains a significant gap in knowledge regarding the condition in adolescents and young adults (AYA) (ages 15–39) and adults (ages 40 and above). AYA/adults account for 10–30% of MB and most patients are diagnosed with MB_SHH_, MB_WNT,_ or Group 4 [1, 4, 5]. Treatment approaches for AYA/adult patients with MB are largely extrapolated from pediatric protocols, which may not sufficiently address the unique characteristics of the disease in older patients [6]. Consequently, elucidating the distinct features of MB in AYA and adults is crucial for developing tailored treatment strategies and improving clinical outcomes.

Genetic tumor syndromes such as Gorlin syndrome (*SUFU*, *PTCH1*), Li-Fraumeni syndrome (*TP53*), Familial Adenomatous Polyposis (*APC*), and Fanconi anemia (*BRCA2, PALB2*) have been identified as genetic risk factors for MB in children, adolescents, and young adults [7]. However, most genetic studies in MB have primarily focused on pediatric patients, leaving the role of genetic tumor syndromes in AYA and adult patients with MB largely unexplored. The discovery of novel germline variants associated with AYA/adult MB would have significant implications for genetic counseling and testing, potentially further improving clinical management for patients with MB.

In this study, we present a case of a young adult patient with MB associated with a germline loss-of-function (LOF) variant in the *TRAF2* gene. Furthermore, we conducted a molecular comparison of this patient with our in-house cohort of 19 MB cases, highlighting the unique molecular characteristics of the *TRAF2*-associated MB. To our knowledge, this is the first documented instance of a young adult patient with medulloblastoma harboring a germline *TRAF2* variant. This case underscores the heterogeneity of the disease and the necessity for a deeper exploration of genetic tumor syndromes in the AYA and adult MB population.

## Case presentation

**Fig. 1:**
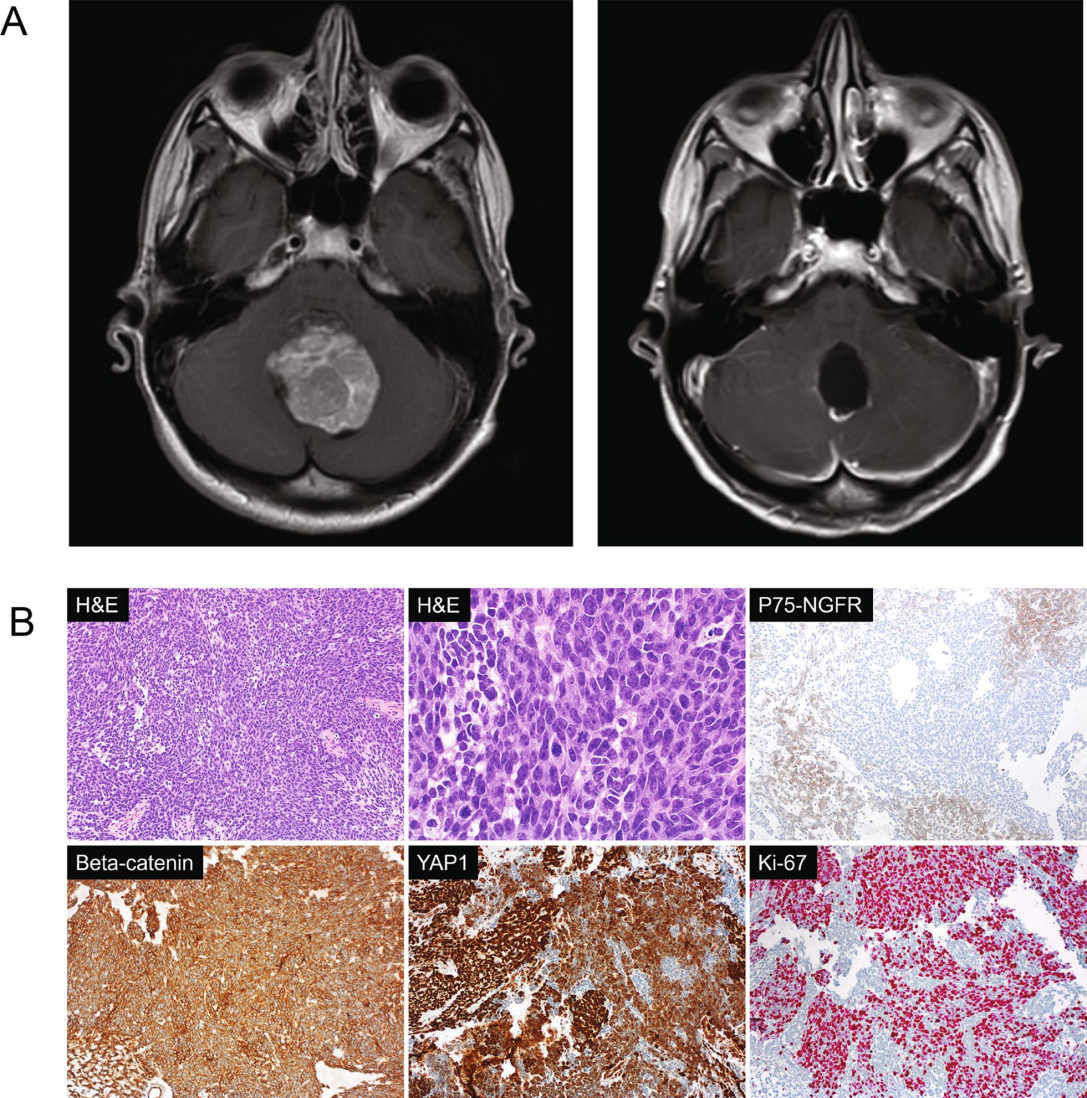
**A** MRI Brain. Left: T1 Axial FLAIR+ gadolinium at diagnosis, revealing large, midline, enhancing posterior fossa mass. Right: Post-operative T1 Axial+ gadolinium, showing evidence of suboccipital craniotomy with surgical cavity without gross residual disease. **B** Histopathology**.** Top left and middle: H&E stained sections show a small round blue cell tumor with brisk mitotic activity; Top right: N75-NGFR immunostain shows patchy positivity; Bottom left: β-catenin immunostain shows membranous positivity in the tumor cells without nuclear accumulation; Bottom middle: YAP1 immunostain shows nuclear and cytoplasmic positivity in the majority of tumor cells; Bottom left: The Ki-67 proliferation index is markedly increased. All images are ×100 magnification except the top middle image which is ×400 magnification

A 21-year-old woman with no significant past medical history presented to her primary care provider with a 3-week history of worsening headaches accompanied by nausea. She also noted difficulty with walking and felt unsteady. She was referred to a local emergency department, where a computed tomography (CT) scan, and brain magnetic resonance imaging (MRI) revealed a 4.5 cm contrast-enhancing mass in the posterior fossa, which caused obstructive hydrocephalus with brainstem compression, tonsillar herniation, and moderate dilation of the third and lateral ventricles (Fig. 1A). An MRI of the spine and cerebrospinal fluid analysis showed no evidence of metastatic disease. Her family history was not significant for malignancy. She underwent a gross total resection (GTR) of the mass via occipital craniectomy complicated by severe posterior fossa syndrome, including mutism, emotional lability, and ataxia. Histological examination of the tumor showed a small round blue cell tumor with brisk mitotic activity. The tumor was positive for synaptophysin, exhibited membranous but not nuclear immunoreactivity for β-catenin, displayed combined nuclear and cytoplasmic positivity for YAP1, was patchy positive for p75-NGFR, and negative for GAB1. The Ki-67 proliferation index was elevated (Fig. 1B**)**. These findings supported the diagnosis of medulloblastoma, classic histology, WHO grade 4. The overall risk classification was deemed "average risk" [8].

**Fig. 2:**
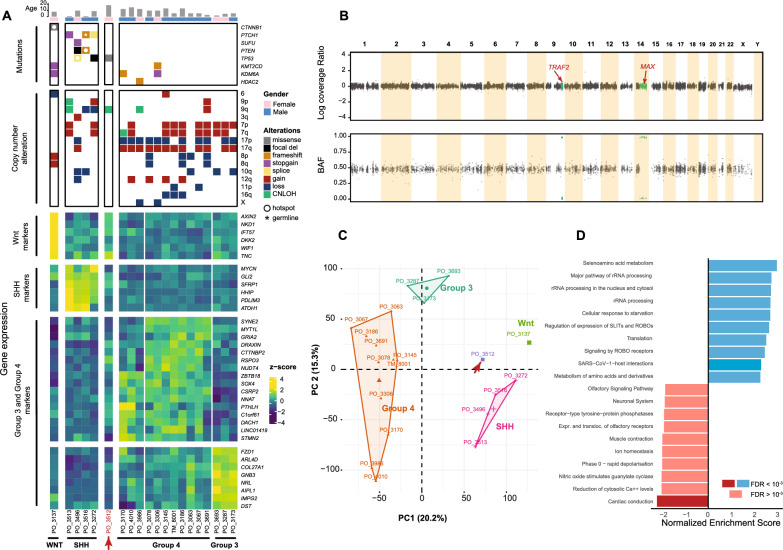
**A** Oncoprint (top half) and gene expression heatmap (bottom half) of 20 medulloblastoma patients. Relevant drivers and cytogenetic events were included for the oncoprint. Gene signatures associated with MB subtypes were curated from a published scRNA-seq study (Materials and methods). Hierarchical clustering using these gene expression markers was employed to guide tentative subgroup assignment (Materials and methods). The arrows indicated the index case, also denoted as PO_3512. **B** Copy number result of our patient. Top: log coverage ratio of tumor versus matches normal; Bottom: B-allelic fraction (BAF) values. The hg19 coordinate of CNLOH segment covering *TRAF2* is chr9:131,909,737–141,213,430. **C** Transcriptome-wide Principal component analysis (PCA) (Materials and methods). The percentages indicate the proportion of variance explained by each principal component. **D** Gene set enrichment analysis (GSEA) was performed on the gene expression data of our patient using the Reactome Pathway Database. The gene list was pre-ranked based on the z-scores calculated from 20 MB cases (Materials and methods)

Next-generation sequencing, including mRNA sequencing and DNA sequencing, was conducted on a formalin-fixed, paraffin-embedded (FFPE) tissue block and matched peripheral blood (Materials and methods). In silico estimation revealed the tumor content to be very high (97%). Somatic mutations are presented in Table 1. We further conducted subsequent analyses of the patient’s MB genome in conjunction with genomes from 19 other MB patients at our institution. At first glance, there were some genomic features suggestive of a Sonic hedgehog (SHH)-activated MB (Fig. 2A). The tumor harbored a copy-neutral loss-of-heterozygosity (LOH) of 9q (Fig. 2B), typically seen in the MB_SHH_ subgroup and most frequent in the MB_SHH-α_ and MB_SHH-δ_ subtypes [9]. In addition, the presence of a somatic *MAX* hotspot mutation (R60Q) (Table 1) was consistent with MB_SHH_, as *MAX* hotspot mutations have previously been observed exclusively in the MB_SHH_ subgroup (in two adults and one child) [10].

**Table 1:** Somatic and rare germline variants detected with target gene panel sequencing in our index case

Gene	Position (in hg19)	Status	Effect	AA change	dbSNP	Normal VAF (%)	Tumor VAF (%)	CADD	1000G Frequency (%)
*TP53*	chr17:7577121	Somatic	Missense	R273C	rs121913343	0	48	27.7	0
*MAX*	chr14:65544747	Somatic	Missense	R60Q		0	96	21.3	0
*MYC*	chr8:128750684	Somatic	Missense	P74L		0	39	22.7	0
*HTR1A*	chr5:63256991	Somatic	Missense	A186T	rs557845021	0	6	21.7	0
*SMAD1*	chr4:146436129	Somatic	Missense	I122F		0	45	27.2	0
*MAD1L1*	chr7:1855841	Somatic	Missense	K674N		0	54	22.1	0
*CHD7*	chr8:61765674	Somatic	Frameshift	F2131fs		0	48		0
*BIRC2*	chr11:102239036	Somatic	Frameshift	I376fs		0	48		0
*ERBB3*	chr12:56495522	Somatic	Frameshift	T1238fs		0	45		0
*NPPB*	chr1:11918519	Germline	Missense	R47H	rs5229	50	42	4.388	0.38
*BCAR3*	chr1:94048471	Germline	Missense	S358N	rs142961077	48	49	18.79	0.12
*CACNA1S*	chr1:201047035	Germline	Missense	R531C	rs751671175	45	52	35	0
*HOXD11*	chr2:176972168	Germline	Missense	A29S	rs149509317	48	44	22.2	0.30
*SP110*	chr2:231037654	Germline	Missense	E538K	rs149682257	49	48	11.77	0.02
*ROBO2*	chr3:77611868	Germline	Missense	V502L	rs562416957	47	44	23.3	0.08
*TET2*	chr4:106156969	Germline	Missense	T624A		48	50	0.015	0
*NEK1*	chr4:170359272	Germline	Missense	S881C	rs543224510	52	47	10.48	0.10
*FAT1*	chr4:187628746	Germline	Missense	T746A	rs372906523	46	47	16.15	0.20
*FGFR4*	chr5:176523696	Germline	Missense	R703W	rs774539398	45	42	20.4	0
*NSD1*	chr5:176637800	Germline	Missense	M800I	rs747267793	48	46	0.001	0
*MAML1*	chr5:179192601	Germline	Missense	H197L	rs558589991	48	49	9.29	0.06
*HIST1H1E*	chr6:26156758	Germline	Missense	A47V	rs141942142	43	51	16.62	0.06
*TRAF3IP2*	chr6:111912641	Germline	Missense	P226T	rs139282334	52	46	18.82	0.26
*TRAF3IP2*	chr6:111913185	Germline	Inframe Insertion	P44dup	rs142054894	49	47	18.72	0
*SLC22A1*	chr6:160543233	Germline	Missense	R89K	rs371964408	52	46	4.019	0
*TBP*	chr6:170871046	Germline	Inframe Deletion	Q91_Q94del		36	36	22	0
*GLI3*	chr7:42004286	Germline	Missense	I1462T	rs371464477	51	50	8.522	0
*FGFR1*	chr8:38273475	Germline	Missense	H589Q		49	45	21.8	0
*TSC1*	chr9:135786868	Germline	Missense	S334L	rs118203481	46	99	21	0.02
***TRAF2***	**chr9:139818372**	**Germline**	**Stopgain**	**R403X**		**45**	**99**	**39**	**0**
*CYP2C9*	chr10:96740981	Germline	Missense	R335W	rs28371685	48	43	25.5	0.72
*ABCC2*	chr10:101590486	Germline	Missense	G921S	rs41318029	47	46	0.27	0.34
*TEAD4*	chr12:3104098	Germline	Missense	A56T	rs148347828	46	48	32	0.02
*BAZ2A*	chr12:56992943	Germline	Missense	R1793Q	rs762971471	52	52	28.6	0
*LATS2*	chr13:21562482	Germline	Inframe Insertion	A478_P479dup	rs550642106	72	65	11.88	0
*BRCA2*	chr13:32918788	Germline	Missense	D2312V	rs80358916	50	41	27	0.02
*TINF2*	chr14:24709952	Germline	Missense	S245Y	rs142777869	47	43	14.6	0.10
*SOS2*	chr14:50585300	Germline	Missense	T1254R		52	97	10.78	0
*MC1R*	chr16:89986546	Germline	Missense	D294H	rs1805009	48	45	20.9	0.34
*SMYD4*	chr17:1686759	Germline	Missense	Q678K	rs530674372	42	51	6.053	0.48
*GID4*	chr17:17962266	Germline	Missense	V231I	rs200321621	46	52	22.4	0
*CD79A*	chr19:42383204	Germline	Missense	T75M	rs199967393	46	48	8.94	0.02
*ASXL1*	chr20:31022796	Germline	Missense	A761T	rs146052718	46	51	25.4	0
*DNMT3B*	chr20:31375172	Germline	Missense	R190H	rs774138031	48	47	10.64	0
*DNMT3B*	chr20:31383238	Germline	Missense	A384T	rs150682895	51	46	14.35	0.54
*ZMYND8*	chr20:45867566	Germline	Inframe Insertion	Q847dup	rs569370037	40	40	14.86	0
*CECR2*	chr22:18031793	Germline	Missense	P1288L	rs181553013	40	42	14.82	0.08
*BCR*	chr22:23653975	Germline	Frameshift	V1094fs	rs372013175	24	14	37	0
*ZNRF3*	chr22:29444445	Germline	Missense	H227Q	rs55775472	48	49	26.1	0.18
*BRWD3*	chrX:79978103	Germline	Missense	K612E		47	49	22.6	0

DNA methylation-based tumor classification of the index tumor was performed using two brain tumor classifiers (Materials and methods). The Heidelberg-DKFZ brain tumor classifier revealed a calibrated score of 0.837 that the tumor belongs MB_SHH_ class, falling short of the 0.90 score required for definitive MB subgroup classification. The NCI (Bethesda) brain tumor classifier identified the tumor as a neuroblastic embryonal tumor with a high score of 0.992. However, it was unable to definitively determine the molecular subtype. The classifier's best match was MB_SHH-δ_, though this was associated with a low score of 0.253. Moreover, the tumor showed no significant mRNA expression of the curated markers associated with the four MB subgroups (Materials and methods, Fig. 2A). Principal component analysis of the tumor's global gene expression profile was inconclusive based on the first two principal components (Fig. 2C). Together, these findings suggested a medulloblastoma with atypical molecular features.

Other notable genomic findings included a heterozygous somatic *MYC* hotspot mutation (P74L) located within the MYC Box 1 phospho-degron, which enhances the stability of MYC by affecting protein degradation [11]. The somatic *MAX* hotspot mutation (R60Q) was homozygous via copy-neutral LOH of chromosome 14 (Table 1, Fig. 2B). Given that MYC and MAX form a heterodimer [12], double hits in *MYC* and *MAX* likely contributed to dysregulation of MYC signaling. In our patient, *MYC* expression was significantly elevated, ranking second among 20 samples in the MB cohort with exceeding levels relative to MB Group 3, where *MYC* overexpression is a known driver [9, 10]. Although no focal *MYC* amplification was observed, this finding could imply a possible structural rearrangement that affected *MYC* enhancer that may not have been detected by targeted panel sequencing. Gene set enrichment analysis further demonstrated that several upregulated genes in this tumor, relative to the rest of the MB cohort, were enriched in pathways related to RNA processing and translation (Fig. 2D). This pattern aligns with the known role of MYC in enhancing RNA processing and translation [13], suggesting that MYC signaling contributed to MB development in our patient.

*Partial* copy-neutral LOH of 9q is a less frequent cytogenetic event in MB_SHH_ (Fig. 2B). The LOH region did not encompass *PTCH1* and *ELP1*, two driver genes commonly affected by 9q LOH events in MB_SHH_ [10, 14]. A detailed analysis of all protein-coding genes within the 9q34.3 LOH region revealed a rare germline loss-of-function (LOF) variant in *TRAF2* (p.Arg403Ter; c.1207C > T). No other rare germline LOF variants were identified in the 9q LOH region, apart from a rare, clinically benign germline missense variant in *TSC1* (p.S334L; ClinVar VCV000041686). We further confirmed that the *TRAF2* LOF variant is heterozygous in the patient’s germline genome (VAF 45%) and homozygous in the MB genome (VAF 99%) (Table 1). Further familial genetic testing was not possible; hence, we could not ascertain parental inheritance. We were, however, able to determine that *TRAF2* p.Arg403Ter is exceedingly rare in humans with a germline carrier frequency of 1 in 806,922 (gnomAD database v4.1.0).

**Fig. 3:**
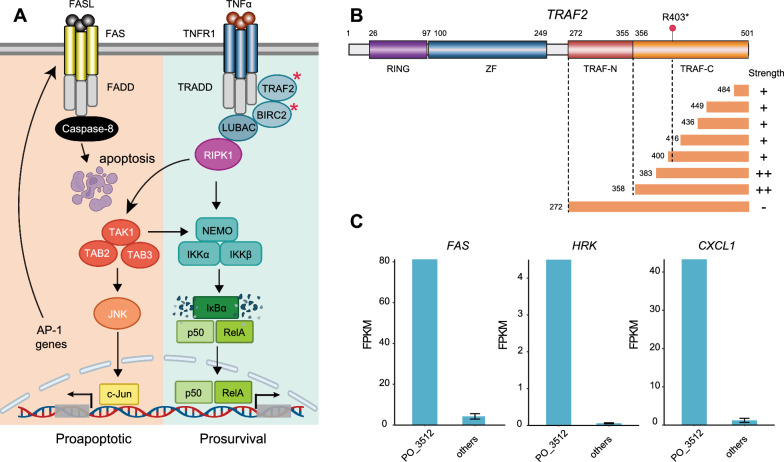
**A** TRAF2 is involved in TNF-mediated NF-κB and JNK signaling activation, which could result in proliferation and apoptosis. **B** Top: Domain structure of TRAF2 protein. The germline variant was depicted as the red dot. Bottom: various TRAF2 mutants and their NF-kB dependent luciferase expression: +, tenfold over the level seen in the vector control; ++, a greater than 20-fold induction over the level seen in the vector control. Light orange boxes denote deleted regions. The figure was adapted from Takeuchi et al*.*, Journal of Biological Chemistry, 1996 [21]. **C** Gene expression in Fragments Per Kilobase of transcript per Million mapped reads (FPKM)

Tumor necrosis factor (TNF) receptor-associated factor 2 (TRAF2) is a ubiquitin ligase integral to various signaling pathways in innate immunity, inflammation, or apoptosis (Fig. 3A) [15, 16]. In pro-survival pathways, TRAF2 functions as a positive regulator of TNF-mediated signaling. Upon TNF binding to TNF receptor 1 (TNFR1), TRAF2 is recruited to TNFR1 by interacting with the adaptor protein TRADD and Receptor Interacting Protein 1 (RIPK1). Subsequently, TRAF2 recruits Cellular Inhibitor of Apoptosis 1 and 2 proteins (cIAP1/2, also known as BIRC2/3), which facilitates the polyubiquitination of RIPK1 and other components of the signaling complex. Ubiquitinated RIPK1 interacts with TGFβ-activated kinase 1 (TAK1) and other proteins to activate the canonical nuclear factor-κB (NF-κB) signaling pathway (Fig. 3A), resulting in the expression of various genes involved in proliferation and inflammation [15, 16]. Additionally, TRAF2 participates in TNF-mediated activation of the c-Jun N-terminal kinase (JNK) signaling pathway through RIPK1 and TAK1 (Fig. 3A) [16–18]. The JNK signaling pathway can induce Activator Protein 1 (AP-1) dependent gene expression. This signaling cascade can also lead to apoptosis, although the precise mechanisms remain unknown [17].

Like other members of the TRAF family, TRAF2 possesses an N-terminal domain characterized by a RING structure and four zinc fingers [19]. The C-terminal TRAF domain is subdivided into the TRAF-N and TRAF-C subdomains. The TRAF-N subdomain features a coiled-coil structure that facilitates both homo- and hetero-oligomerization. The TRAF-C subdomain consists of antiparallel β-strands and interacts with receptor and adaptor proteins, including BIRC2 [20]. The stopgain variant identified in this patient significantly truncates the TRAF-C subdomain, thus potentially affecting its capacity to bind with other crucial signaling proteins (Fig. 3B).

However, it has been observed that the TRAF2 protein with a partially truncated C-terminal domain may still retain some residual binding activity [21]. In a functional study, various mutants with progressively truncated TRAF-C domains were engineered and overexpressed in HEK293 cells [21]. These synthetic deletions demonstrated the ability to activate the downstream signaling pathway to varying degrees (Fig. 3B). One of their constructs truncated *TRAF2* at codon 400, closely resembling the truncation at codon 403 due to the germline LOF variant described in our index patient. Truncation of *TRAF2* at codon 400 exhibited a milder activation of downstream signaling compared to the construct that completely eliminated the TRAF-C domain (Fig. 3B). Strikingly, our patient further harbors a heterozygous somatic LOF mutation in the E3 ligase *BIRC2* (Table 1). In this context, the somatic LOF *BIRC2* mutation converges on the same pathway, further diminishing the residual binding efficacy of TRAF2, thereby potentially leading to additional inhibition of apoptosis (Fig. 3A).

Transcriptome provided deeper insights into the oncogenic context of the signaling pathway involving *TRAF2* and *BIRC2*. Our patient exhibited significantly higher expression levels of pro-apoptotic genes, such as Fas receptor (*FAS*) and harakiri (*HRK*) [22, 23], compared to other tumors in our MB cohort (Fig. 3C). The tumor also demonstrated elevated expression level of chemokine (C-X-C motif) ligand 1 (*CXCL1*), which is linked to inflammation [24]. These findings lead us to hypothesize that an inflammation-like phenotype could have contributed to  tumorigenesis. Subsequently, biallelic loss of *TRAF2* and monoallelic loss of *BIRC2* could have collaborated in evading apoptosis and thus triggering MB development.

## Discussion and conclusion

Despite the advancements facilitated by next-generation sequencing in elucidating the genomic diversity of medulloblastoma, our understanding of this complex disease remains incomplete. MB in AYA and adult patients introduces additional complexity due to its unique genomic and epigenomic features, which differ from pediatric cases. Group 3 medulloblastoma is rarely observed in adults. Approximately 60% of adult medulloblastoma cases are SHH-activated, 25% belong to Group 4, and 15% are WNT-activated [4, 5]. MB_SHH_ is further divided into four subtypes with distinct molecular profiles and clinical outcomes. Among them, MB_SHH-δ_ is the most common in young adults and adults, with a median age of onset of 26 years, and have better prognosis [4, 9].

Our 21-year-old patient with MB presents a challenge to the current central nervous system (CNS) tumor classification system. Immunohistochemistry revealed that the tumor lacked nuclear β-catenin, but showed both nuclear and cytoplasmic YAP1 positivity, as well as N75-NGFR, supporting its classification as MB_SHH_ [25]. However, DNA methylation classification and gene expression clustering results remained inconclusive. Additionally, the tumor lacked somatic mutations in *PTCH1*, *SUFU*, *SMO*, and the *TERT* promoter, which are typically seen in MB_SHH_ [1, 9, 10]. Also, somatic and germline *TP53* mutations are generally found in MB_SHH_ patients aged 5–18 years [26]. Furthermore, *MYC* overexpression is more commonly associated with MB Group 3 rather than MB_SHH_ [1, 9, 10].

MB has been linked to various genetic tumor syndromes. Gorlin syndrome, which arises from germline variants in *PTCH1* and *SUFU*, is associated with an increased risk of the desmoplastic/nodular subtype of medulloblastoma. Li-Fraumeni syndrome, characterized by germline variants in *TP53*, increases susceptibility to medulloblastoma and a range of other malignancies. *APC*-associated polyposis increases the risk of medulloblastoma, particularly the WNT subgroup [7, 27]. Recently, pathogenic germline variants in *BRCA2*, *PALB2*, *GPR161*, and *ELP1* have also been implicated in medulloblastoma malignancy [7, 14, 28]. It has been estimated that 40% of pediatric patients with MB_SHH_ harbor a genetic tumor syndrome [14]. The case presented in our study suggests that *TRAF2*-associated medulloblastoma is a novel genetic tumor syndrome.

Somatic mutations in *TRAF2* are observed in less than 6% of human cancers [29]. Somatic loss-of-function mutations and deletions in *TRAF2* have been identified as drivers in hematological malignancies, including mantle cell lymphoma, diffuse large B-cell lymphoma, and multiple myeloma [30, 31]. This highlights the critical role of TRAF2 as a negative regulator of the non-canonical NF-κB pathway in lymphoid cells [30, 31]. However, the role of TRAF2 in solid tumors remains largely unexplored, further complicated by its dual function as both an anti-apoptotic and pro-apoptotic factor within a complex cellular signaling network [15–18]. In the context of CNS tumors, another member of the TRAF family and E3 ubiquitin ligase, *TRAF7*, has been identified as a somatic driver in meningioma [32].

In addition to the germline *TRAF2* loss-of-function variant and partial copy-neutral LOH of 9q, this patient’s tumor also acquired a somatic LOF mutation in *BIRC2*, another E3 ubiquitin ligase that interacts with TRAF2. Similar to TRAF2, BIRC2 has been identified as a tumor suppressor in hematological malignancies [31], while it may function as an oncogene in other tumors through gene amplification [33]. *BIRC2* and *TRAF2* potentially act as tumor suppressors within a signaling axis that typically induces cell death, such as through JNK-induced apoptosis or TNF-stimulated necrosis [17, 18]. Notably, somatic hotspot mutations in another E3 ligase, *KBTBD4*, have been characterized in MB [34]. Our findings could have significant implications for understanding the broader role of dysregulated E3 ubiquitin ligases in medulloblastoma.

In conclusion, our case report suggests that germline loss-of-function *TRAF2 *alterations may represent a novel risk factor for medulloblastoma. Further studies in adolescent, young adult, and adult MB populations are warranted to determine their prevalence, full spectrum of co-occurring somatic driver alterations, and therapeutic and prognostic implications.

## Treatment outcomes

The patient received craniospinal irradiation (CSI) treatment in accordance with standard-of-care protocols. She was administered a dose of 23.4 Gy to the entire neuraxis and an additional 30.6 Gy boost to the tumor bed in 30 fractions, accompanied by weekly intravenous vincristine. Furthermore, the patient underwent chemotherapy as per the Children's Oncology Group protocol ACNS0331. This regimen includes cisplatin, CCNU, vincristine, and cyclophosphamide, administered as maintenance cycles over a total of seven cycles. Maintenance chemotherapy was discontinued prematurely for this patient due to profound and prolonged cytopenias. During the course of therapy, the patient developed bilateral high-frequency hearing loss at 8000 and 12,000 Hz, necessitating a reduction in the cisplatin dose. The patient has been disease-free for 5 years and 4 months following surgery. Annual MRI brain and spine scans will continue for surveillance purposes. The patient and her family have been informed about the rarity of the germline variant and its potential association with hematological malignancies.

## Materials and methods

Clinical sequencing was conducted following standard protocols in our Clinical Laboratory Improvement Amendments (CLIA)-certified sequencing laboratory [35]. For each patient, we performed targeted sequencing on DNA from the tumor and matched normal peripheral blood, using a panel covering 1,714 genes, including probes for the *TERT* promoter region. Tumor RNA sequencing was performed with an exome-capture transcriptome protocol developed in our lab, covering all coding genes. Bioinformatic analyses—including mutation calling, copy number analyses, and gene expression profiling—were carried out as previously described [35].

Gene expression signatures were derived from a published single-cell RNA sequencing (scRNA-seq) study, which performed UMAP clustering on 39,946 single cells from 28 MB patients [36]. For the WNT, SHH, and Group 3 clusters, the top 10 genes with the highest log fold change from differential expression analyses (as described in [36]) were selected. For Group 4, the top 20 genes were chosen due to the high heterogeneity within this group. Lowly expressed genes were removed to ensure reliable detection in bulk sequencing data, resulting in a final set of 36 expression markers (Fig. 2A). Hierarchical clustering, based on Euclidean distance and the single-linkage method, was applied to the log2-transformed Fragments Per Kilobase of transcript per Million mapped reads (FPKM) values. This clustering approach identified tentative WNT, SHH, Group 3, and Group 4 subgroups, with the index patient identified as an outlier (Fig. 2A heatmap). Genomic features, including mutations and copy number alterations, were curated from previous genomic studies and reviews [1, 9, 10], and aligned with RNA-seq-derived subgroup classifications (Fig. 2A Oncoprint).

Additionally, transcriptome-wide principal component analysis (PCA) was performed on the log-transformed FPKM values. Gene variance was calculated across samples for each gene’s log-transformed expression data. Low-variance genes were filtered by removing the bottom 10% with the lowest variance, based on variance quantiles (Fig. 2C). Gene set enrichment analysis of the index case, using a pre-ranked list of z-scores calculated among 20 patients, was performed on the Reactome Pathways Database with WebGestalt (Fig. 2D).

DNA methylation analysis of the index case was performed using the Infinium MethylationEPIC v2.0 Kit. DNA methylation-based tumor classification of the index case was conducted with the Heidelberg-DKFZ brain tumor classifier v12.5 (https://www.molecularneuropathology.org/mnp) and the NCI (Bethesda) classifier v2.

## Data Availability

All sequencing data (FASTQ and BAM files) and methylation array data are available upon reasonable request.

## References

[1] Northcott PA, Robinson GW, Kratz CP, Mabbott DJ, Pomeroy SL, Clifford SC, Rutkowski S, Ellison DW, Malkin D, Taylor MD et al (2019) Medulloblastoma. Nat Rev Dis Primers 5:11 30765705 10.1038/s41572-019-0063-6

[2] Chevignard M, Câmara-Costa H, Doz F, Dellatolas G (2017) Core deficits and quality of survival after childhood medulloblastoma: a review. Neurooncol Pract 4:82–97 31385962 10.1093/nop/npw013 PMC6655396

[3] Schwalbe EC, Lindsey JC, Nakjang S, Crosier S, Smith AJ, Hicks D, Rafiee G, Hill RM, Iliasova A, Stone T et al (2017) Novel molecular subgroups for clinical classification and outcome prediction in childhood medulloblastoma: a cohort study. Lancet Oncol 18:958–971 28545823 10.1016/S1470-2045(17)30243-7 PMC5489698

[4] Wooley JR, Penas-Prado M (2021) Pediatric versus adult medulloblastoma: towards a definition that goes beyond age. Cancers. 10.3390/cancers13246313 34944933 10.3390/cancers13246313 PMC8699201

[5] Franceschi E, Giannini C, Furtner J, Pajtler KW, Asioli S, Guzman R, Seidel C, Gatto L, Hau P (2022) Adult medulloblastoma: updates on current management and future perspectives. Cancers. 10.3390/cancers14153708 35954372 10.3390/cancers14153708 PMC9367316

[6] Wong GC-H, Li KK-W, Poon MF-M, Ng H-K (2020) Is adult medulloblastoma merely the counterpart of pediatric medulloblastoma? Glioma 3:90

[7] Waszak SM, Northcott PA, Buchhalter I, Robinson GW, Sutter C, Groebner S, Grund KB, Brugières L, Jones DTW, Pajtler KW et al (2018) Spectrum and prevalence of genetic predisposition in medulloblastoma: a retrospective genetic study and prospective validation in a clinical trial cohort. Lancet Oncol 19:785–798 29753700 10.1016/S1470-2045(18)30242-0 PMC5984248

[8] Louis DN, Perry A, Wesseling P, Brat DJ, Cree IA, Figarella-Branger D, Hawkins C, Ng HK, Pfister SM, Reifenberger G et al (2021) The 2021 WHO classification of tumors of the central nervous system: a summary. Neuro Oncol 23:1231–1251 34185076 10.1093/neuonc/noab106 PMC8328013

[9] Cavalli FMG, Remke M, Rampasek L, Peacock J, Shih DJH, Luu B, Garzia L, Torchia J, Nor C, Morrissy AS et al (2017) Intertumoral heterogeneity within medulloblastoma subgroups. Cancer Cell 31:737-754.e6 28609654 10.1016/j.ccell.2017.05.005 PMC6163053

[10] Northcott PA, Buchhalter I, Morrissy AS, Hovestadt V, Weischenfeldt J, Ehrenberger T, Gröbner S, Segura-Wang M, Zichner T, Rudneva VA et al (2017) The whole-genome landscape of medulloblastoma subtypes. Nature 547:311–317 28726821 10.1038/nature22973 PMC5905700

[11] Schaub FX, Dhankani V, Berger AC, Trivedi M, Richardson AB, Shaw R, Zhao W, Zhang X, Ventura A, Liu Y et al (2018) Pan-cancer alterations of the MYC oncogene and its proximal network across the cancer genome atlas. Cell Syst 6:282-300.e2 29596783 10.1016/j.cels.2018.03.003 PMC5892207

[12] Cascón A, Robledo M (2012) MAX and MYC: a heritable breakup. Cancer Res 72:3119–3124 22706201 10.1158/0008-5472.CAN-11-3891

[13] Cole MD, Cowling VH (2008) Transcription-independent functions of MYC: regulation of translation and DNA replication. Nat Rev Mol Cell Biol 9:810–815 18698328 10.1038/nrm2467 PMC3880805

[14] Waszak SM, Robinson GW, Gudenas BL, Smith KS, Forget A, Kojic M, Garcia-Lopez J, Hadley J, Hamilton KV, Indersie E et al (2020) Germline elongator mutations in sonic hedgehog medulloblastoma. Nature 580:396–401 32296180 10.1038/s41586-020-2164-5 PMC7430762

[15] Borghi A, Verstrepen L, Beyaert R (2016) TRAF2 multitasking in TNF receptor-induced signaling to NF-κB, MAP kinases and cell death. Biochem Pharmacol 116:1–10 26993379 10.1016/j.bcp.2016.03.009

[16] Brenner D, Blaser H, Mak TW (2015) Regulation of tumour necrosis factor signalling: live or let die. Nat Rev Immunol 15:362–374 26008591 10.1038/nri3834

[17] Dhanasekaran DN, Reddy EP (2008) JNK signaling in apoptosis. Oncogene 27:6245–6251 18931691 10.1038/onc.2008.301 PMC3063296

[18] Ventura J-J, Cogswell P, Flavell RA, Baldwin AS Jr, Davis RJ (2004) JNK potentiates TNF-stimulated necrosis by increasing the production of cytotoxic reactive oxygen species. Genes Dev 18:2905–2915 15545623 10.1101/gad.1223004 PMC534651

[19] Yin Q, Lamothe B, Darnay BG, Wu H (2009) Structural basis for the lack of E2 interaction in the RING domain of TRAF2. Biochemistry 48:10558–10567 19810754 10.1021/bi901462e PMC2830148

[20] Park HH (2018) Structure of TRAF family: current understanding of receptor recognition. Front Immunol 9:1999 30214450 10.3389/fimmu.2018.01999 PMC6125299

[21] Takeuchi M, Rothe M, Goeddel DV (1996) Anatomy of TRAF2: distinct domains for nuclear factor-ΚB activation and association with tumor necrosis factor signaling proteins. J Biol Chem 271:19935–19942 8702708 10.1074/jbc.271.33.19935

[22] Strasser A, Jost PJ, Nagata S (2009) The many roles of FAS receptor signaling in the immune system. Immunity 30:180–192 19239902 10.1016/j.immuni.2009.01.001 PMC2956119

[23] Coultas L, Terzano S, Thomas T, Voss A, Reid K, Stanley EG, Scott CL, Bouillet P, Bartlett P, Ham J et al (2007) Hrk/DP5 contributes to the apoptosis of select neuronal populations but is dispensable for haematopoietic cell apoptosis. J Cell Sci 120:2044–2052 17535852 10.1242/jcs.002063 PMC2795636

[24] Zhou C, Gao Y, Ding P, Wu T, Ji G (2023) The role of CXCL family members in different diseases. Cell Death Discov 9:212 37393391 10.1038/s41420-023-01524-9 PMC10314943

[25] Pietsch T, Haberler C (2016) Update on the integrated histopathological and genetic classification of medulloblastoma—a practical diagnostic guideline. Clin Neuropathol 35:344–352 27781424 10.5414/NP300999 PMC5094373

[26] Zhukova N, Ramaswamy V, Remke M, Pfaff E, Shih DJH, Martin DC, Castelo-Branco P, Baskin B, Ray PN, Bouffet E et al (2013) Subgroup-specific prognostic implications of TP53 mutation in medulloblastoma. J Clin Orthod 31:2927–2935 10.1200/JCO.2012.48.5052 PMC4878050 23835706

[27] Carta R, Del Baldo G, Miele E, Po A, Besharat ZM, Nazio F, Colafati GS, Piccirilli E, Agolini E, Rinelli M et al (2020) Cancer predisposition syndromes and medulloblastoma in the molecular era. Front Oncol 10:566822 33194646 10.3389/fonc.2020.566822 PMC7658916

[28] Begemann M, Waszak SM, Robinson GW, Jäger N, Sharma T, Knopp C, Kraft F, Moser O, Mynarek M, Guerrini-Rousseau L et al (2020) Germline GPR161 mutations predispose to pediatric medulloblastoma. J Clin Oncol 38:43–50 31609649 10.1200/JCO.19.00577 PMC6943973

[29] Zhu S, Jin J, Gokhale S, Lu AM, Shan H, Feng J, Xie P (2018) Genetic alterations of TRAF proteins in human cancers. Front Immunol 9:2111 30294322 10.3389/fimmu.2018.02111 PMC6158389

[30] Siegmund D, Wagner J, Wajant H (2022) TNF receptor associated factor 2 (TRAF2) signaling in cancer. Cancers. 10.3390/cancers14164055 36011046 10.3390/cancers14164055 PMC9406534

[31] Vo JN, Wu Y-M, Mishler J, Hall S, Mannan R, Wang L, Ning Y, Zhou J, Hopkins AC, Estill JC et al (2022) The genetic heterogeneity and drug resistance mechanisms of relapsed refractory multiple myeloma. Nat Commun 13:1–13 35768438 10.1038/s41467-022-31430-0 PMC9243087

[32] Clark VE, Erson-Omay EZ, Serin A, Yin J, Cotney J, Ozduman K, Avşar T, Li J, Murray PB, Henegariu O et al (2013) Genomic analysis of non-NF2 meningiomas reveals mutations in TRAF7, KLF4, AKT1, and SMO. Science 339:1077–1080 23348505 10.1126/science.1233009 PMC4808587

[33] Cancer Genome Atlas Network (2015) Comprehensive genomic characterization of head and neck squamous cell carcinomas. Nature 517:576–582 25631445 10.1038/nature14129 PMC4311405

[34] Chen Z, Ioris RM, Richardson S, Van Ess AN, Vendrell I, Kessler BM, Buffa FM, Busino L, Clifford SC, Bullock AN et al (2022) Disease-associated KBTBD4 mutations in medulloblastoma elicit neomorphic ubiquitylation activity to promote CoREST degradation. Cell Death Differ 29:1955–1969 35379950 10.1038/s41418-022-00983-4 PMC9525703

[35] Robinson DR, Wu Y-M, Lonigro RJ, Vats P, Cobain E, Everett J, Cao X, Rabban E, Kumar-Sinha C, Raymond V et al (2017) Integrative clinical genomics of metastatic cancer. Nature 548:297–303 28783718 10.1038/nature23306 PMC5995337

[36] Riemondy KA, Venkataraman S, Willard N, Nellan A, Sanford B, Griesinger AM, Amani V, Mitra S, Hankinson TC, Handler MH et al (2022) Neoplastic and immune single-cell transcriptomics define subgroup-specific intra-tumoral heterogeneity of childhood medulloblastoma. Neuro Oncol 24:273–286 34077540 10.1093/neuonc/noab135 PMC8804892

